# Non-commutative structures of brain and cognition: quantum and contextual probability as a translational framework

**DOI:** 10.3389/fnhum.2026.1882287

**Published:** 2026-07-17

**Authors:** Haruki Emori, Andrei Khrennikov, Atsushi Iriki

**Affiliations:** 1 Graduate School of Information Science and Technology, Hokkaido University, Sapporo, Japan; 2 RIKEN, Center for Interdisciplinary Theoretical and Mathematical Sciences (iTHEMS), Wako, Japan; 3 Advanced Comprehensive Research Organization, Teikyo University, Tokyo, Japan; 4 Institute of Mathematics and Physics, Linnaeus University, Växjö, Sweden

**Keywords:** cognitive structural science, contextual probability, neural oscillations, non-commutative probability, order effect, quantum cognition

## Abstract

Cognitive neuroscience has accumulated robust findings (e.g., order effects in judgment, multi-path motor preparation, perceptual binding, attentional selection, and the recursive construction of self and time) that systematically resist explanation within classical probabilistic and causal frameworks. We argue that these are not anomalies but signatures of a deeper, non-commutative architecture of cognition and brain dynamics. We propose quantum probability theory together with contextual probability theory, not as metaphorical analogies but as rigorous translational languages for cognitive processes in which observation actively transforms underlying state spaces. Underlying this framework is the conjecture that neural network dynamics in the brain are intrinsically organized to generate quantum-like representations—rather than merely being described by quantum mathematics from the outside. Their structural primitives (i.e., superposition, entanglement, projection, and non-commutativity) map onto premotor population coding, long-range cortical synchrony, prefrontal state dynamics, and default-mode network activity. On this basis we sketch a new sub-domain (namely, Cognitive Structural Science) that treats the geometry and algebra of cognitive state spaces as primary explananda, and outline a research program combining homologous human–macaque experiments with quantum-computer simulation as a constrained testbed.

## 1 Introduction

Cognitive neuroscience has come of age as a quantitative science. Decades of psychophysics, neuroimaging, and electrophysiology have made operational the once-philosophical questions of perception, decision, memory, and self. And yet, an awkward residue persists. Robust phenomena have been documented that classical probabilistic and causal models cannot accommodate without *ad hoc* machinery: judgments depend on the order in which questions are asked (Wang et al., 2014); the premotor cortex represents multiple action plans in parallel before commitment (Cisek and Kalaska, 2005); distributed cortical activity generates a unified percept whose factorization into components is operationally elusive (Singer, 1999; Fries, 2015); attention does not merely amplify but appears to constitute what is perceived (Dehaene and Changeux, 2011); and the self that recalls is recursively bound to the self being recalled (Northoff, 2016). These observations are usually managed in isolation, each confined to its own task literature, each treated as noise, bias, or boundary phenomenon. Their cumulative weight, however, suggests that they share a common architectural feature.

Our claim is structural. The five families share the property that observation (judgment, attention, recall) does not simply read out a pre-existing state but actively transforms the state space within which subsequent observations are defined. This is precisely the condition under which Kolmogorov-style probability ceases to be the appropriate descriptive language. The mathematics that does carry this property natively was developed for quantum mechanics, but its informational content is not exclusive to physics. As Khrennikov et al. have argued (Khrennikov, 2009, 2010; Busemeyer and Bruza, 2012; Pothos and Busemeyer, 2013; Khrennikov et al., 2025a), the same formalism can be redeployed as a translational language for cognition, not because the brain is a quantum physical system, but because cognitive state transitions exhibit the very algebraic non-commutativity, contextual probability-space construction, and measurement-induced state change that the formalism was built to handle. Our use of quantum vocabulary is therefore mathematical, not ontological: it picks out the structural features that a Hilbert-space model captures and a sample-space model does not.

This Perspective develops three claims. First (Section 2), the five empirical signatures form a coherent family unified by non-commutative structural primitives (superposition, entanglement, projection, and non-commutativity) each with a documented neural correlate. Second (Section 3), quantum probability and contextual probability (Khrennikov, 2009, 2010) provide the translational mathematics, with explicit physiological grounding via the correspondence between neural oscillatory covariance and density operators. Third (Section 4), we propose a new sub-domain, Cognitive Structural Science, and outline a comparative research program across humans, non-human primates, and quantum-computer simulation. We close (Section 5) with a brief reflection on what this re-description buys, and what it does not.

A few words on framing are in order. The position we take is neither the strong ontological claim that the brain implements quantum mechanics at biological scales, nor the weak claim that quantum vocabulary is a useful metaphor. Both miss what the formalism actually offers, which is a mathematics of state spaces in which observation is constitutive rather than passive. Cognitive science already deals routinely with such observation-constitutive operations (judgment, attention, recall, reframing) and the case to be made is empirical: are the deviations from classicality structured in the way the formalism predicts, and does its physiological grounding via oscillatory covariance yield testable predictions that classical accounts cannot make? Crucially, our conjecture is not that quantum mathematics merely fits cognitive data, but that neural network organization in the brain is functionally structured so as to generate quantum-like informational states as an intrinsic architectural outcome. This conjecture is continuous with prior open-system and generalized-probability formulations of quantum-like modeling in biology, cognition, decision-making, and neuronal networks (Khrennikov, 2023; Khrennikov et al., 2025b). This distinguishes the present approach from quantum cognition as a modeling toolkit: the target of investigation is not merely the fit of a formalism but the structural source of that fit in neural dynamics.

## 2 Five non-classical signatures of cognition and brain

**Figure 1:**
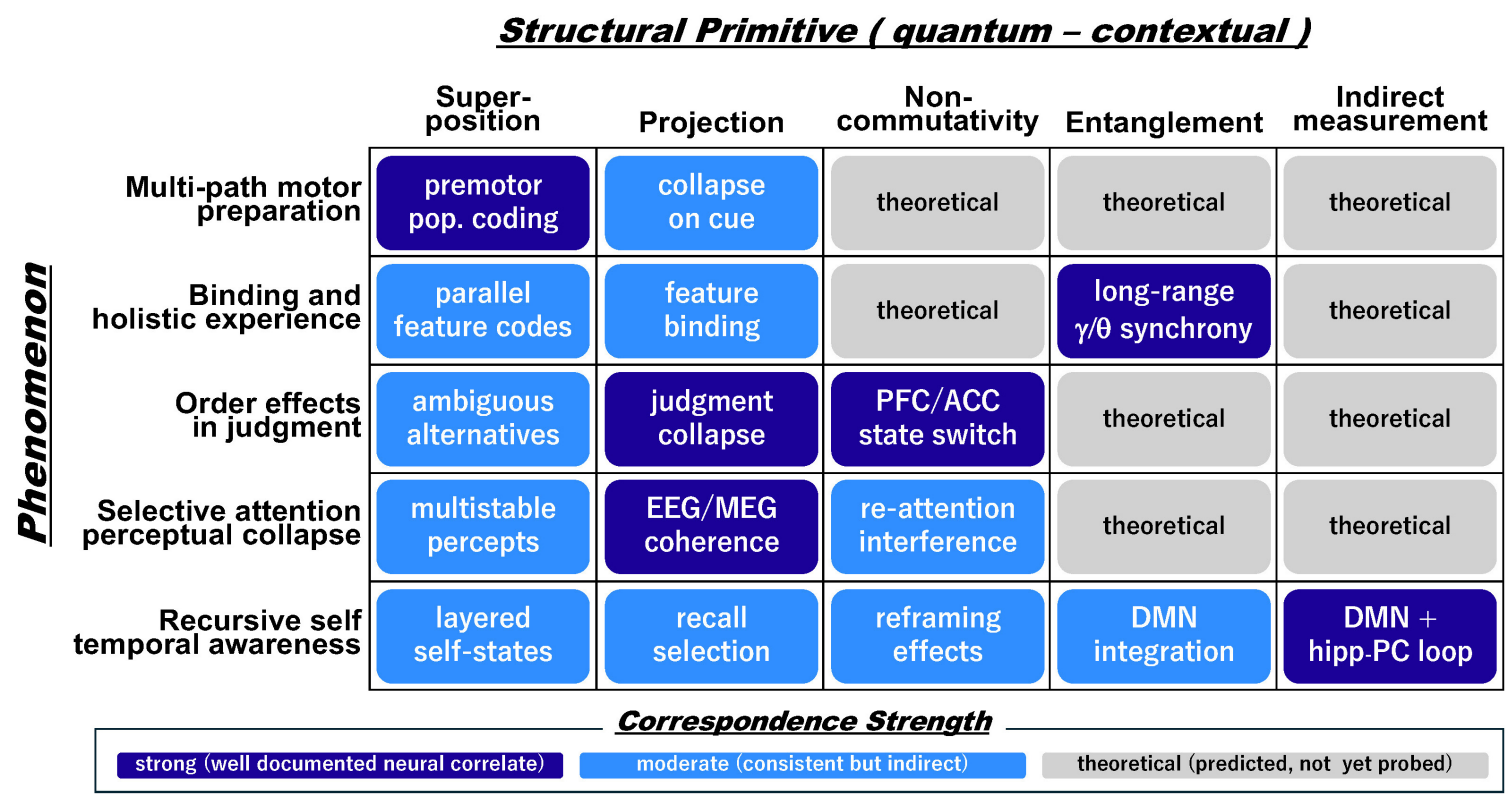
Structural primitive–phenomenon–substrate matrix for five non-classical signatures of cognition. Columns indicate structural primitives (superposition; projection; non-commutativity; entanglement; indirect measurement); rows indicate empirical phenomena (multi-path motor preparation; binding and holistic experience; order effects in judgment; selective attention; recursive self). Cell shading encodes correspondence strength: dark blue, strong (well-documented neural correlate); light blue, moderate (consistent but indirect); gray, theoretical (predicted, not yet directly probed). The candidate neural substrate for each phenomenon is labeled within its column: premotor population coding; long-range gamma/theta synchrony; prefrontal–anterior cingulate state dynamics; attentional EEG/MEG coherence reorganization; default-mode–hippocampal–prefrontal circuit activity.

For each phenomenon below we summarize the observation, indicate where the classical framework strains, name the structural primitive that recovers the regularity, and point to the neural substrate that anchors the description in physiology. The five are neither exhaustive nor isolated; they are chosen because each, on its own, has been subject to rigorous empirical work, and because together they sample motor, perceptual, decisional, attentional, and self-referential processes. Figure 1 assembles the resulting structural primitive–phenomenon–substrate correspondence in matrix form.

### 2.1 Multi-path motor preparation as superposition

Single-unit and population recordings in primate dorsal premotor cortex demonstrate that, before the disambiguating cue arrives, neural populations simultaneously represent more than one candidate movement (Cisek and Kalaska, 2005; Dekleva et al., 2018). The observed activity is not a graded probabilistic mixture from which one trajectory is later sampled; it is a structured coexistence of action plans that interfere and ultimately collapse into one. Classical race or drift-diffusion models fit choice latencies but require independent accumulators, which miss the explicit interference structure that population dynamics actually display. The corresponding formal object is a state vector |ψ〉 = α|L〉 + β|R〉, and the Feynman path-integral idiom (amplitudes summed across paths weighted by an action-like cost) captures the convergence dynamics directly. The biological lesson is that motor preparation is not *post-hoc* commitment but pre-cued superposition. Crucially, the predicted interference between candidate paths can be probed by manipulating the relative cost or salience of each option mid-trial, and tracking whether the population trajectory shows constructive or destructive shifts that go beyond the simple weighting of independent accumulators.

### 2.2 Binding as structural entanglement

The unified character of conscious perception across distributed cortical regions has resisted reduction to mere synchrony (Singer, 1999; Fries, 2015). Long-range gamma and theta coherence are necessary but not, on their own, sufficient: what they implement is a structurally non-factorizable composite state. In formal terms, this corresponds to entanglement: a composite state ρ_*AB*_ whose partial traces ρ_*A*_ and ρ_*B*_ fail to reconstruct the whole system. Through Bell inequalities adapted to neural and behavioral data (Conte et al., 2009; Khrennikov, 2010; Asano et al., 2015), this empirically predicts violations of factorability conditions that additive synchrony measures cannot capture. The signature is operational: composite responses to conjoined stimuli should exceed the bound permitted by any locally separable model.

Where such violations occur, the case for treating binding as a structural rather than merely communicative phenomenon becomes stronger. The same logic extends from classical sensory binding to higher-order cognitive processes (such as empathy, the recognition of another's intentions, or the felt unity of a remembered scene) where elements drawn from distant cortical territories combine into experiences that resist clean decomposition (Tononi, 2004).

Recent work has begun to clarify how entanglement-like structures can emerge in neuronal networks without invoking literal quantum physics. Khrennikov and Yamada (2025) introduced a Hilbert-space representation of neuronal activity in which distributed assemblies generate joint informational states whose statistical structure cannot be decomposed into independent local contributions, by modeling these as “mental entanglement.” Related quantum-probabilistic analysis of hierarchical neural processing has been developed in Khrennikov et al. (2025a). Taken together, these approaches suggest that binding may reflect a deeper non-local organization of information processing in the brain, and that quantum-like formalism provides its natural mathematical language. Consistent with this, quantum-inspired analysis of EEG using a p-adic quantum potential (Shor et al., 2021) demonstrated high sensitivity in distinguishing healthy subjects from patients with depression, schizophrenia, and cognitive decline, illustrating how structural entanglement measures may serve as diagnostic indicators of the integrity of cognitive information processing.

### 2.3 Order effects as non-commuting measurement

When two judgments A and B are elicited in different orders, response distributions systematically violate classical commutativity, P(A∩B) ≠ P(B∩A) (Wang et al., 2014), where P(A∩B) denotes the sequential joint probability P(A)·P(B|A), which is well defined in quantum probability even though the classical conjunction of non-commuting events is not. The QQ-equality (a parameter-free relation among such sequential joint probabilities, derived within the quantum formalism as a falsifiable signature of quantum-like structure) is corroborated across attitude, perception, and memory tasks (Pothos and Busemeyer, 2022). The result is striking because the prediction is not fitted: the model contains no free parameter to absorb a fit failure. Neurally, prefrontal and anterior cingulate networks exhibit order-sensitive state transitions (Mante et al., 2013), consistent with the action of non-commuting projection operators on a shared state.

A more fine-grained formal treatment is provided by the theory of quantum instruments (Ozawa and Khrennikov, 2021, 2023; Khrennikov, 2023), which distinguishes non-commutativity at the level of observables from non-commutativity at the level of the state transformers that measurements induce—a separation of direct relevance for modeling compound psychological effects in which question order interacts with response replicability (Fuyama et al., 2025). Order effects are therefore not biases to be corrected away; they are evidence that the operations of judgment, considered as algebraic objects, do not commute. What follows methodologically is that the temporal order of probes (across questions, tasks, or trials) must be treated as a structural feature of experimental design rather than a nuisance to be counterbalanced away.

### 2.4 Selective attention as projective measurement

Studies of ambiguous figures (Conte et al., 2009; Asano et al., 2015), binocular rivalry (Blake and Logothetis, 2002), and inattentional blindness (Simons and Chabris, 1999) establish that attention does not merely modulate the gain of pre-existing percepts; it constitutes which possibility becomes definite. This is the structural role of projective measurement: |ψ〉 $\to \hat{\text{P}}$_a|ψ〉/||$\hat{\text{P}}$_a|ψ〉||, with the Born rule supplying the probabilities and an interference signature in re-attended trials. Cortical EEG/MEG correlates (rapid coherence reorganization at the locus of the attended feature) match the abruptness predicted by projection rather than the smooth integrative dynamics expected under classical filtering (Fries, 2015). Crucially, the formalism predicts that re-attention to a previously suppressed feature should leave history-dependent traces in the response distribution, an interference fingerprint that classical attentional gain models do not generate. The translation that this provides is concrete: a perceptual decision is not a noisy report on a fixed scene but a projection of a structured potential into a definite actuality, with the act of projection leaving an algebraic trace that subsequent observations can detect.

### 2.5 The recursive self as indirect measurement

Self-experience is doubled: a subjective self that observes, and an objective self that is observed and reported. Autobiographical recall in particular is constructive, contextually re-evaluated, and routed through default-mode and hippocampal–prefrontal circuits (Henson and Gagnepain, 2010; Northoff, 2016). Quantum mechanics provides a precise structural template for this layering: indirect measurement, where the observer interacts with the system only through a probe whose final read-out reveals system properties without direct contact. The objective self serves as the probe; recall is its measurement (Ozawa, 1984). This unifies temporal layering, reframing effects, and the dynamic stability of identity within one formal pattern, and it predicts that manipulations of the probe (reframing, third-person perspective, evaluative load) will produce structured, not arbitrary, shifts in the recovered self-state. Read this way, the recursive self is not an embarrassment for cognitive theory but a paradigmatic instance of the kind of architecture in which the present mathematics is designed to work.

## 3 Quantum and contextual probability as a translational language

### 3.1 Why classical probability falls short

Kolmogorov probability presumes a fixed event space having the Boolean structure, additivity over disjoint events, and a single conditional structure. In cognition, none of these is dependable. The event space against which a judgment is evaluated is itself reshaped by prior judgments; conditional probabilities depend on the route, not only the destination; and the same stimulus, embedded in different contexts, can recruit incompatible decision lattices that cannot be merged into one Boolean algebra. These are not features of merely noisy classical processes, but they are categorical departures from the classical scheme, and they accumulate fastest in precisely the high-level phenomena we sampled in Section 2.

### 3.2 Core structures of quantum probability

Quantum probability theory replaces the event space with a Hilbert space H. Cognitive states are vectors |Ψ〉∈H or, more generally, density operators ρ (Aerts, 2009; Khrennikov, 2009, 2010; Busemeyer and Bruza, 2012). Observables (perceptual categories, response options, memory retrieval contexts) correspond to self-adjoint operators with spectral projections {$\hat{\text{P}}$_a}; the probability of outcome a is given by the Born rule, p(a) = 〈ψ|$\hat{\text{P}}$_a|ψ〉, or Tr(ρ $\hat{\text{P}}$_a). When two observables Â, B^ fail to commute, [Â, B^] ≠ 0, the order of measurements matters: the same input state yields different joint statistics under A-then-B vs. B-then-A. This is the order effect, expressed in algebra rather than *ad hoc*. Importantly, the framework introduces interference terms (cross-products between projection amplitudes) that are systematically absent from classical mixtures. Where empirical distributions carry these terms, the case for non-commutative description is constructive rather than exclusionary. None of this requires the brain to compute Hilbert-space operations; what is required is that the joint statistics of behavior and neural response inherit a structure that classical sample-space models cannot reproduce without enlarging their event space *ad hoc*.

### 3.3 Contextual probability and the oscillatory neural substrate

The contextual probability theory generalizes the framework: each act of judgment instantiates its own probability space, and shifts in context (priming, instruction, attentional set, emotional valence) are not perturbations of a common space but constructors of new ones (Khrennikov, 2009; Atmanspacher, 2015; Khrennikov and Basieva, 2014). This layer connects naturally to neural physiology. As recently formalized (Khrennikov et al., 2025a), populations of neuronal oscillators with random phase relations admit representation as complex-valued probability variables; their covariance structure plays the role of a density operator. Context shifts (instructional changes, attentional reorientation, emotional re-weighting) appear, on this view, as reconfigurations of oscillatory covariance, that is, of ρ. Aperiodic and oscillatory components recently parsed in human electrophysiology (Donoghue et al., 2020) provide a candidate observational handle on this reconfiguration. The translation from formal structure to physiology, in this account, is not metaphorical but operational: density operators are not merely analogous to oscillatory states; they are, given the correspondence, the appropriate algebraic encoding of those states. This shift carries weight: it removes the lingering charge that quantum vocabulary in cognitive science is interpretive license, and replaces it with a specific, falsifiable mapping between an observable physiological quantity and a formal object whose properties (positivity, trace-class behavior, transformation rules under unitary evolution) have measurable consequences. Empirically, the most immediate handle is the statistical structure of pairwise oscillator covariance during context shifts: classical predictions and density-operator predictions diverge in their cross-frequency coupling and re-organization speed.

### 3.4 Operationalizing non-commutativity: path integrals and Leggett–Garg inequalities

To rigorously operationalize this framework, the temporal evolution of cognitive states can be formalized via a Feynman path integral over the configuration space of neural oscillatory dynamics (Iriki and Tanaka, 2024). This approach allows for the analytical derivation of multi-time correlation functions, *C*(*t*_*i*_, *t*_*j*_), between sequential cognitive observations. Under the strict assumption of classical macro-realism, these temporal correlation functions are fundamentally bounded by Leggett-Garg inequalities (Leggett and Garg, 1985); the dynamic, temporal equivalent of Bell inequalities (e.g., *C*(*t*_1_, *t*_2_)+*C*(*t*_2_, *t*_3_)−*C*(*t*_1_, *t*_3_) ≤ 1). However, the hallmark of quantumness in any physical or informational system manifests structurally as the non-commutativity of its observables or operations. If cognitive observation actively transforms the underlying state space, sequential measurement operators will fundamentally fail to commute (i.e., [Â(*t*_*i*_), Â(*t*_*j*_)]≠0). It is precisely this non-commutativity that mathematically precludes the existence of a joint, measurement-independent probability distribution. Within the path integral formalism, this non-commutative algebraic structure necessitates the superposition of complex probability amplitudes across alternative state trajectories, inherently generating interference terms that predict systematic violations of the classical Leggett-Garg bounds. Consequently, evaluating these path-integral-derived correlation inequalities against empirical neural time-series provides a parameter-free, mathematically definitive criterion for experimentally verifying the non-commutative structural signatures of macroscopic brain dynamics.

### 3.5 Process-based non-classicality and indefinite causal order

An adjacent line of work, the Vienna research program on indefinite causal order and causally non-separable processes associated with Brukner and collaborators, provides a useful conceptual parallel (Oreshkov et al., 2012; Costa and Shrapnel, 2016). Its central lesson is that non-classical probabilistic structures can arise from the organization of operations and transformations, rather than from microscopic physical constituents alone. In the process-matrix framework, a process is causally separable when it can be decomposed as a probabilistic mixture of the orders A before B and B before A; when no such decomposition exists, the order of operations is indefinite rather than merely unknown.

The quantum switch makes this point experimentally concrete by coherently controlling the order of two operations, so that AB and BA contribute within a single information-processing architecture (Procopio et al., 2015; Rubino et al., 2017; Rozema et al., 2024). We do not suggest that neural systems physically implement quantum switches or process matrices. The relevance is methodological and structural: it shows that non-classical behavior may be generated at the level of process organization. This strengthens, without ontologizing, the present claim that contextuality, order effects, and related cognitive phenomena should be studied as properties of cognitive operations and their compositional structure.

## 4 Toward cognitive structural science: a programmatic outlook

### 4.1 The proposal

We name the resulting research program “*Cognitive Structural Science*.” It is not the claim that the brain is a quantum physical device. It is the proposal that cognitive neuroscience adopt, as a first-class theoretical commitment, the geometry and algebra of cognitive state spaces, how they are structured, when they shift, under what observations, and with what compositional rules. Where the dominant tradition asks which state was selected, structural science asks on what kind of space the selection unfolded. This shift is methodological as well as substantive: it reorients model building toward operations that can change the state space itself, rather than only the trajectories within it.

### 4.2 Discriminating quantum from quantum-like in empirical data

The distinction developed in Sections 3.4 and 3.5 directly motivates the experimental agenda. Order-effect paradigms refined to test the QQ-equality probe non-commutativity of observables. Cross-modal binding tasks, designed to compare additive synchrony predictions with non-factorizability conditions, probe entanglement-like structure. Attention–rivalry paradigms with sequential re-attention probe the non-commutativity of projections. Each can be specified as a test of a discriminating inequality; failure of the inequality in one direction implicates the non-commutativity of observables, in another, the non-commutativity of operations, and in a third, fully classical mixture (with appropriate corrections). The key methodological commitment is to prefer experimental designs whose three possible outcomes are themselves theoretically informative, rather than designs that merely fit one model. Practically, this means re-running canonical paradigms with two changes: embedding the design within a triple of conditions tailored to the discriminating inequality, and recording neural correlates dense enough to track state reorganization during, not only after, context shifts.

### 4.3 A comparative agenda: humans, non-human primates, and quantum simulators

A cognitive structural science is testable across species. Aperiodic and oscillatory dynamics in human EEG/MEG, neuroimaging-based cortical state estimation, and homologous tasks in macaques with single-unit and population recordings provide a graded substrate from molecular timescales to behavior. Quantum computers, used as constrained simulators rather than as biological claims, offer an additional dimension: they natively realize the high-dimensional non-commutative state spaces the hypotheses predict, allowing direct model comparison among classical, generative, and quantum-inspired accounts on identical empirical datasets. This triadic approach (human neurophysiology, primate neurophysiology, and constrained quantum simulation) extends the logic of triadic niche construction (Iriki and Tanaka, 2024) into a falsifiable comparative neuroscience of context-sensitive state transitions, of the kind that suitable multi-laboratory programs should be sought to deliver. The combination is necessary precisely because each strand answers a question the others cannot: human electrophysiology fixes the structural claims to clinically relevant temporal scales; macaque neurophysiology grants causal and cellular resolution; quantum simulation provides a counterfactual environment in which model spaces can be probed at sizes intractable on classical hardware.

### 4.4 What this re-description does not claim

We do not claim ontological quantum-ness for the brain. We also do not claim that neural systems instantiate the quantum switch or any specific process-matrix model of indefinite causal order. We do not claim that all cognitive phenomena require quantum probability, much of cognition is well described classically (Friston, 2010; Clark, 2013), and the program's value depends on locating where, and how, classicality fails. We do not claim that violations arising from non-commutative observables and those from non-commutative operations are equivalent; their dissociation is part of the empirical agenda. The program's virtue, instead, is that it converts what has long been treated as residue into structural signal, and provides a single mathematical lingua franca in which physics-of-information, computational neuroscience, and cognitive science can talk to each other. We are, in other words, proposing a re-coordination of vocabularies, not a takeover (Khrennikov, 2010; Bruza et al., 2015; Khrennikov et al., 2025a).

## 5 Conclusion

Order effects, multi-path preparation, binding, attentional collapse, and the recursive self are not five independent puzzles. They are, we have argued, members of a single family: non-commutative signatures of a cognitive architecture in which observation transforms state. Related work on indefinite causal order reinforces the same operational lesson: non-classical probabilistic behavior can arise from the organization of transformations without being reduced to the microscopic state of a single constituent. Quantum probability and contextual extension supply the translational mathematics; the correspondence between neural oscillatory covariance and density operators supplies the physiological anchor; and the dissociation between observable and operational non-commutativity supplies the falsifiability the program needs to be scientific rather than rhetorical. We propose that Cognitive Structural Science be developed in parallel with, not in opposition to, the existing classical apparatus, and that its central question (on what kind of space does cognition operate) become a routine target of empirical investigation. The next experiments to perform are the ones whose outcomes the present languages cannot predict. If the deviations we have cataloged resolve, under suitable inequalities, into clear classes of observable or operational non-commutativity, the field will have inherited a genuinely new descriptive layer; if they do not, we will have, at minimum, sharpened the boundary where classical descriptions remain sufficient. Either way, the question moves from vocabulary to evidence.

## Data Availability

The original contributions presented in the study are included in the article, further inquiries can be directed to the corresponding author.

## References

[B1] Aerts D. (2009). Quantum structure in cognition. J. Math. Psychol. 53, 314–348. doi: 10.1016/j.jmp.2009.04.005

[B2] Asano M. Khrennikov A. Ohya M. Tanaka Y. Yamato I. (2015). Quantum Adaptivity in Biology: From Genetics to Cognition. Berlin: Springer. doi: 10.1007/978-94-017-9819-8

[B3] Atmanspacher H. (2015). Contextual emergence of mental states. Cogn. Process. 16, 359–364. doi: 10.1007/s10339-015-0658-0 26018611

[B4] Blake R. Logothetis N. K. (2002). Visual competition. Nat. Rev. Neurosci. 3, 13–21. doi: 10.1038/nrn701 11823801

[B5] Bruza P. D. Wang Z. Busemeyer J. R. (2015). Quantum cognition: a new theoretical approach to psychology. Trends Cogn. Sci. 19, 383–393. doi: 10.1016/j.tics.2015.05.001 26058709

[B6] Busemeyer J. R. Bruza P. D. (2012). Quantum Models of Cognition and Decision. Cambridge: Cambridge University Press. doi: 10.1017/CBO9780511997716

[B7] Cisek P. Kalaska J. F. (2005). Neural correlates of reaching decisions in dorsal premotor cortex: specification of multiple direction choices and final selection of action. Neuron 45, 801–814. doi: 10.1016/j.neuron.2005.01.027 15748854

[B8] Clark A. (2013). Whatever next? Predictive brains, situated agents, and the future of cognitive science. Behav. Brain Sci. 36, 181–204. doi: 10.1017/S0140525X12000477 23663408

[B9] Conte E. Khrennikov A. Todarello O. Federici A. Mendolicchio L. Zbilut J. P. (2009). Mental states follow quantum mechanics during perception and cognition of ambiguous figures. Open Syst. Inf. Dyn. 16, 85–100. doi: 10.1142/S1230161209000074

[B10] Costa F. Shrapnel S. (2016). Quantum causal modelling. New J. Phys. 18:063032. doi: 10.1088/1367-2630/18/6/063032

[B11] Dehaene S. Changeux J. P. (2011). Experimental and theoretical approaches to conscious processing. Neuron 70, 200–227. doi: 10.1016/j.neuron.2011.03.018 21521609

[B12] Dekleva B. M. Kording K. P. Miller L. E. (2018). Single reach plans in dorsal premotor cortex during a two-target task. Nat. Commun. 9:3556. doi: 10.1038/s41467-018-05959-y 30177686 PMC6120937

[B13] Donoghue T. Haller M. Peterson E. J. Varma P. Sebastian P. Gao R. . (2020). Parameterizing neural power spectra into periodic and aperiodic components. Nat. Neurosci. 23, 1655–1665. doi: 10.1038/s41593-020-00744-x 33230329 PMC8106550

[B14] Fries P. (2015). Rhythms for cognition: communication through coherence. Neuron 88, 220–235. doi: 10.1016/j.neuron.2015.09.034 26447583 PMC4605134

[B15] Friston K. (2010). The free-energy principle: a unified brain theory? Nat. Rev. Neurosci. 11, 127–138. doi: 10.1038/nrn2787 20068583

[B16] Fuyama M. Khrennikov A. Ozawa M. (2025). Quantum-like cognition and decision-making in the light of quantum measurement theory. Phil. Trans. Royal Soc. A 383:20240372. doi: 10.1098/rsta.2024.0372 PMC12658452 41306041

[B17] Henson R. N. Gagnepain P. (2010). Predictive, interactive multiple memory systems. Hippocampus 20, 1315–1326. doi: 10.1002/hipo.20857 20928831

[B18] Iriki A. Tanaka S. (2024). Potential of the path integral and quantum computing for the study of humanities: an underlying principle of human evolution and the function of consciousness. Glob. Perspect. 5:115651. doi: 10.1525/gp.2024.115651

[B19] Khrennikov A. (2009). Contextual Approach to Quantum Formalism. Dordrecht: Springer. doi: 10.1007/978-1-4020-9593-1

[B20] Khrennikov A. (2010). Ubiquitous Quantum Structure: From Psychology to Finance. Berlin: Springer. doi: 10.1007/978-3-642-05101-2

[B21] Khrennikov A. (2023). Open systems, quantum probability, and logic for quantum-like modeling in biology, cognition, and decision-making. Entropy 25:886. doi: 10.3390/e25060886 37372230 PMC10296982

[B22] Khrennikov A. Basieva I. (2014). Possibility to agree on disagree from quantum information and decision making. J. Math. Psychol. 62–63, 1–15. doi: 10.1016/j.jmp.2014.09.003

[B23] Khrennikov A. Iriki A. Basieva I. (2025a). Constructing a bridge between functioning of oscillatory neuronal networks and quantum-like cognition along with quantum-inspired computation and AI. BioSystems. 257:105573. doi: 10.1016/j.biosystems.2025.105573 40889614

[B24] Khrennikov A. Ozawa M. Benninger F. Shor O. (2025b). Coupling quantum-like cognition with the neuronal networks within generalized probability theory. J. Math. Psychol. 125:102923. doi: 10.1016/j.jmp.2025.102923

[B25] Khrennikov A. Yamada M. (2025). Quantum-like representation of neuronal networks' activity: modeling “mental entanglement”. Front. Hum. Neurosci. 19:1685339. doi: 10.3389/fnhum.2025.1685339 41446506 PMC12722948

[B26] Leggett A. J. Garg A. (1985). Quantum mechanics versus macroscopic realism: Is the flux there when nobody looks? Phys. Rev. Lett. 54, 857–860. doi: 10.1103/PhysRevLett.54.857 10031639

[B27] Mante V. Sussillo D. Shenoy K. V. Newsome W. T. (2013). Context-dependent computation by recurrent dynamics in prefrontal cortex. Nature 503, 78–84. doi: 10.1038/nature12742 24201281 PMC4121670

[B28] Northoff G. (2016). Is the self a higher-order or fundamental function of the brain? The basis-model of self-specificity. Cogn. Neurosci. 7, 203–222. doi: 10.1080/17588928.2015.1111868 26505808

[B29] Oreshkov O. Costa F. Brukner C. (2012). Quantum correlations with no causal order. Nat. Commun. 3:1092. doi: 10.1038/ncomms2076 23033068 PMC3493644

[B30] Ozawa M. (1984). Quantum measuring processes of continuous observables. J. Math. Phys. 25, 79–87. doi: 10.1063/1.526000

[B31] Ozawa M. Khrennikov A. (2021). Modeling combination of question order effect, response replicability effect, and QQ-equality with quantum instruments. J. Math. Psychol. 100:102491. doi: 10.1016/j.jmp.2020.102491

[B32] Ozawa M. Khrennikov A. (2023). Nondistributivity of human logic and violation of response replicability effect in cognitive psychology. J. Math. Psychol. 112:102739. doi: 10.1016/j.jmp.2022.102739

[B33] Pothos E. M. Busemeyer J. R. (2013). Can quantum probability provide a new direction for cognitive modeling? Behav. Brain Sci. 36, 255–274. doi: 10.1017/S0140525X12001525 23673021

[B34] Pothos E. M. Busemeyer J. R. (2022). Quantum cognition. Annu. Rev. Psychol. 73, 749–778. doi: 10.1146/annurev-psych-033020-123501 34546804

[B35] Procopio L. M. Moqanaki A. Araújo M. Costa F. Calafell I. A. Dowd E. G. . (2015). Experimental superposition of orders of quantum gates. Nat. Commun. 6:7913. doi: 10.1038/ncomms8913 26250107 PMC4918346

[B36] Rozema L. A. Strömberg T. Cao H. Guo Y. Liu B.-H. Walther P. (2024). Experimental aspects of indefinite causal order in quantum mechanics. Nat. Rev. Phys. 6, 483–499. doi: 10.1038/s42254-024-00739-8

[B37] Rubino G. Rozema L. A. Feix A. Araújo M. Zeuner J. M. Procopio L. M. . (2017). Experimental verification of an indefinite causal order. Sci. Adv. 3:e1602589. doi: 10.1126/sciadv.1602589 28378018 PMC5365250

[B38] Shor O. Glik A. Yaniv-Rosenfeld A. Valevski A. Weizman A. Khrennikov A. . (2021). EEG p-adic quantum potential accurately identifies depression, schizophrenia and cognitive decline. PLoS ONE 16:e0255529. doi: 10.1371/journal.pone.0255529 34351992 PMC8341571

[B39] Simons D. J. Chabris C. F. (1999). Gorillas in our midst: sustained inattentional blindness for dynamic events. Perception 28, 1059–1074. doi: 10.1068/p281059 10694957

[B40] Singer W. (1999). Neuronal synchrony: a versatile code for the definition of relations? Neuron 24, 49–65. doi: 10.1016/s0896-6273(00)80821-1 10677026

[B41] Tononi G. (2004). An information integration theory of consciousness. BMC Neurosci. 5:42. doi: 10.1186/1471-2202-5-42 15522121 PMC543470

[B42] Wang Z. Solloway T. Shiffrin R. M. Busemeyer J. R. (2014). Context effects produced by question orders reveal quantum nature of human judgments. Proc. Natl. Acad. Sci. USA. 111, 9431–9436. doi: 10.1073/pnas.1407756111 24979797 PMC4084470

